# Front-of-Package Nutrition Labeling and Its Impact on Food Industry Practices: A Systematic Review of the Evidence

**DOI:** 10.3390/nu15112630

**Published:** 2023-06-05

**Authors:** Montserrat Ganderats-Fuentes, Sherry Morgan

**Affiliations:** 1 Perelman School of Medicine, University of Pennsylvania, Philadelphia, PA 19104, USA; 2 Holman Biotech Commons, University of Pennsylvania, Philadelphia, PA 19104, USA

**Keywords:** food labeling, front-of-package labels, food industry, product reformulation, nutrition policies, public health, global health

## Abstract

The obesity epidemic has become a major public health concern globally, and the food supply is a significant driver of this trend. Front-of-package (FOP) labels have been implemented in many countries to encourage healthier food choices. This systematic review aimed to examine the effect of FOP label implementation on food manufacturers’ practices. A comprehensive search of multiple databases was conducted following PRISMA guidelines, identifying 39 relevant articles from 1990 to 2021. The studies indicated that FOP labels conveying intuitive information influenced product reformulation, whereas those with numerical information without specific guidance had no impact on reducing unhealthy nutrients. The most common outcomes were sodium, sugar, and calorie reduction. Mandatory policies reported higher and more consistent effects on product reformulation compared to voluntary approaches. Voluntary FOP labeling resulted in low uptake and tended to be applied to healthier products. Food manufacturers responded to FOP labeling heterogeneously, depending on the label design and type of enforcement. FOP label implementation can reduce nutrients of concern but food manufacturers behave strategically by labeling healthier choices. This review provides recommendations for maximizing the benefits of using FOP labels to prevent obesity, and findings can inform future public health research and policymaking.

## 1. Introduction

Over the last four decades, the prevalence of obesity has tripled worldwide, and over 4 million people die annually from overweight-related diseases [1]. Given that environmental factors, lifestyle preferences, and cultural environment all play significant roles in obesity development [1], a comprehensive approach is required to address this public health problem. A crucial area that deserves attention is the food industry, which is a significant driver of the obesity epidemic [2]. Manufacturers can play an essential role in preventing obesity, for example, by removing or reducing unhealthy nutrients from their products, a process known as product reformulation [3]. Product reformulation has been implemented voluntarily in some countries and enforced in others with positive results. For instance, in 2005, the United Kingdom (UK) government encouraged the food industry to reformulate food products to reduce their salt content, leading to a significant decline in the population’s salt intake [4]. In 2004, Denmark took a different approach by implementing a mandatory food reformulation policy, restricting the content of artificial trans fatty acids (TFA) in certain food ingredients. It is estimated that the policy reduced coronary heart disease deaths by 26.5 per 100.000 people per year from 2004 to 2006 [5].

Consumers can also influence product reformulation by guiding the market supply through their choices and preferences [6]. However, for consumers to demand healthier foods, they need access to nutritional information. While back-of-package (BOP) nutrition labels provide this information, they are often difficult to understand [7] and underused [8]. Consequently, mistaken beliefs about food healthfulness can arise [9,10].

To overcome these issues, front-of-package (FOP) labels were introduced. FOP labels provide easy-to-understand nutritional information, helping consumers make healthier choices [11]. Over 30 countries have endorsed at least one type of FOP label [12]. Depending on the country, FOP labels can be voluntary (e.g., UK, New Zealand, France) or mandatory (e.g., Chile, Israel, Ecuador). Their design can vary in several dimensions, such as color, shape, or method to deliver information [12].

The scientific literature has focused mainly on the effect of FOP nutrition labels on consumers’ understanding [13,14,15], perceptions [16,17], and food purchases [18,19,20,21]. Although these labels can help consumers understand nutrition information and guide them to make healthier choices [13,15,16,18,22], results are mixed about the most effective design. Therefore, it is important to examine how food manufacturers respond to these labels to gain a more comprehensive understanding of their impact.

Recent studies have provided valuable insights into the effects of food labeling on industry practices. For instance, Shangguan et al. conducted a meta-analysis that found a significant decrease in trans fatty acids but no decrease in sodium or sugar after food labeling [23]. Similarly, a narrative review by Roberto et al. reported that the implementation of FOP labels encouraged food producers to reformulate [15]. However, there has been no systematic review focusing on the responses of food manufacturers, particularly those involved in the production of pre-packaged foods with FOP labeling.

Thus, this study aimed to systematically review the evidence surrounding the effect of different FOP label designs and enforcement styles on food manufacturers’ practices. By doing so, this study aims to contribute to a more comprehensive understanding of the effectiveness of FOP labeling in reducing unhealthy nutrients in the food supply.

## 2. Materials and Methods

A systematic literature search was conducted following the Preferred Reporting Items for Systematic Reviews and Meta-Analyses guidelines [24]. Appendix A provides the detailed protocol.

### 2.1. Inclusion Criteria and Exclusion Criteria

This review included publications where the exposure was FOP labeling implemented by a government or non-government organization. All standardized FOP label designs and enforcement types—mandatory or voluntary—were included in the sample. The outcomes of interest were changes in packaged food and beverage product formulations, labeling uptake, and nutritional differences between labeled and unlabeled products. Articles chosen were limited to those in English and Spanish published between 1 January 1990 and 18 November 2021. The starting date was chosen to compare results with a previous meta-analysis conducted in 2014 [23], which included peer-reviewed articles found in the databases search but also any other referenced articles. It also included the grey literature for working papers [9,25].

Studies that met any of the following criteria were excluded from the review: (1) non-nutrient-based FOP labels such as organic, GMO, and country of origin; (2) product-specific industry claims, health or nutrition claims, or non-nutritional information such as alcohol content claims; and (3) studies where the abstract or full text was not available.

### 2.2. Information Sources and Search

This review conducted a search across multiple databases, with a primary focus on English-language databases (PubMed, PAIS Index, Cochrane Library, SCOPUS, ABI/Inform, Google Scholar, among others), supplemented by one Spanish-language database for the search (Scielo). The systematic search was performed on 1 September 2020. However, due to the growing available evidence about the topic, there were additional strategic searches on 9 and 28 May 2021. In addition, search updates on the search engine were activated. For the primarily English language databases, the key terms included were “Nutrition label*”, “Nutrition logo”, “Front of Pack*”, “Food label*”, and “Warning Label*”. Outcomes of interest were not included in the search query because the literature addressed them in multiple ways, such as product reformulation and policy effect, among others, and findings could have been limited. Appendix B contains the search strategy details used for each database. However, PubMed reflects the approach and keyword terms, including:

PubMed: ((((“Nutrition label*”[Title/Abstract]) OR (“Nutrition logo”[Title/Abstract])) OR (“Front of Pack*”[Title/Abstract])) OR (“Food label*”[Title/Abstract])) OR (“Warning Label*”[Title/Abstract]).

### 2.3. Article Review and Study Selection

One investigator searched and screened titles and abstracts for relevance. Two people independently conducted the secondary screening analyzing full-text articles for relevance and eligibility. The reviewers met to resolve any discrepancies within their results.

The literature was summarized using a thematic synthesis framework that identified key findings’ components.

### 2.4. Data Extraction

The types of FOP labels were classified into four main categories: (i) non-interpretative or reductive designs, which are labels that provide numerical information with no specific guidance, judgment, or recommendation; [26] (ii) nutrient-specific designs that specify information and guidance on the content of specific nutrients; (iii) summary designs that measure the overall nutritional quality of food products and assign them a score; and (iv) positive endorsement designs that indicate that a product meets specific standards of healthfulness. The last three categories are interpretative designs that use intuitive information to allow consumers to judge a product’s healthfulness.

Due to substantial heterogeneity in the outcomes and their form of measurement (e.g., nutrition label scanner data, document review, manufacturer’s self-report), included studies did not share the same outcome and measure; therefore, this review was limited to a narrative summary of the literature.

This review was not registered in the International Prospective Register of Systematic Reviews database. This review is not research on human subjects; IRB approval was not sought.

## 3. Results

**Figure 1:**
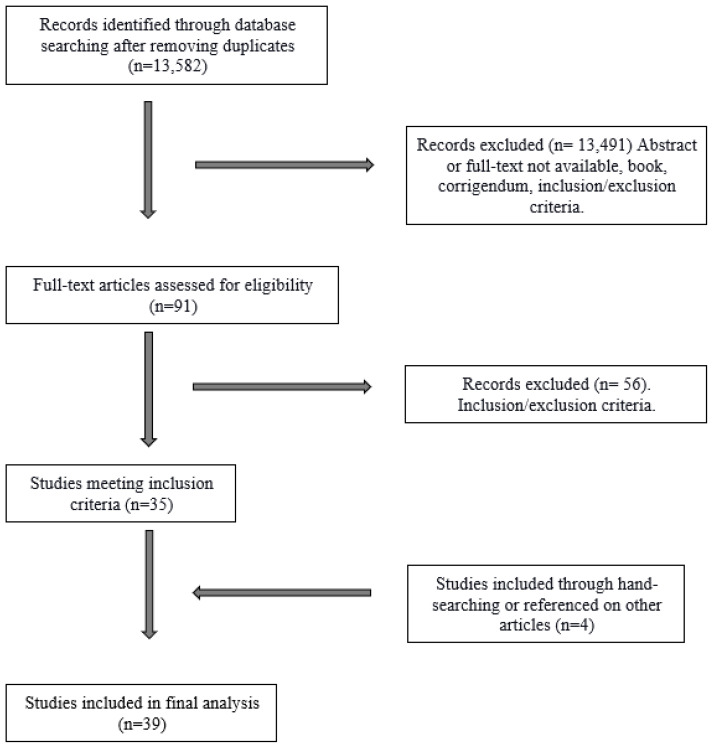
PRISMA flow chart.

Figure 1 represents the process of study selection by a PRISMA flow chart. All citations were imported to Mendeley for initial review. From the initial combined list of 18,037 citations, a title/abstract review of de-duplicated citations was completed using the inclusion/exclusion criteria, resulting in 95 publications. All 95 full articles were screened by two reviewers separately, and four additional articles were identified by hand searching or reviewing the references, resulting in a final citation list of 39 articles suitable for this review.

Table 1 provides descriptive characteristics of the study sample. More than half of the studies evaluated industry responses from Australia and New Zealand (*n* = 21), followed by South America (*n* = 7) and Europe (*n* = 7). A dearth of the literature studied industry practices in other parts of the world. Additionally, the studies represented ten countries and examined 13 label designs. These countries and labels were the sample of analysis for this review.

Table 2 summarizes the FOP nutrition labeling schemes and their corresponding country of implementation and enforcement. In Australia, four label designs were analyzed: two non-interpretive designs (Guideline Daily Amount and energy icon), one summary design (Health Star Rating), and one positive endorsement design (Pick the Tick). Studies from Australia, New Zealand, Europe, and Canada evaluated voluntary labels, while studies in Iran and South America examined mandatory nutrient-specific labeling. The nutrient-specific design was the only one represented in both voluntary and mandatory systems. Lastly, positive endorsement labels were proposed primarily by NGOs or food companies rather than governmental institutions.

**Figure 2:**
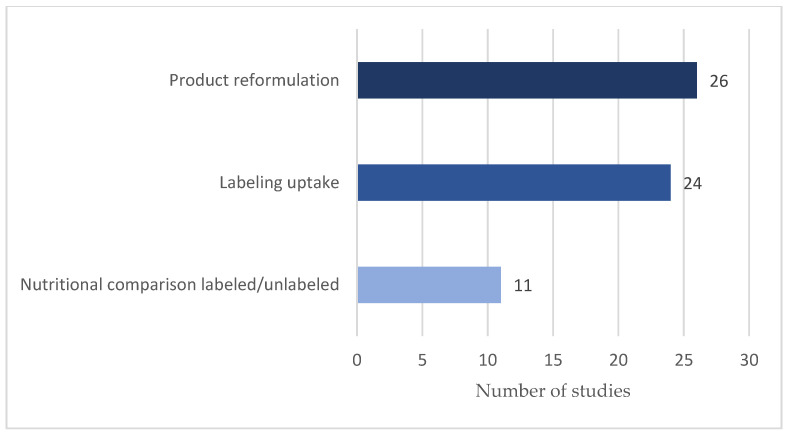
Number of studies by main themes.

**Figure 3:**
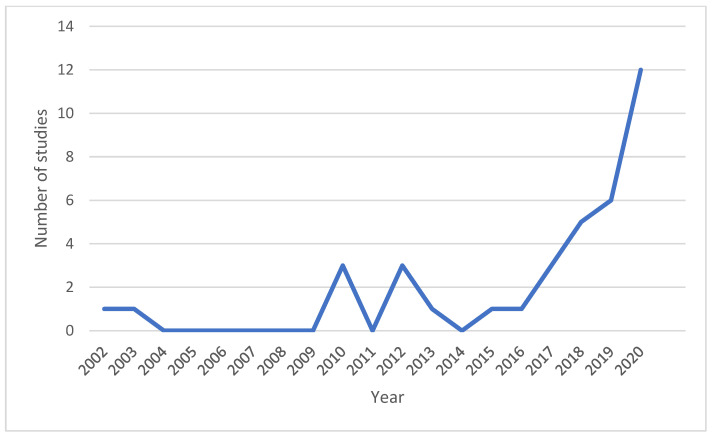
Number of studies by year of publication.

Figure 2 indicates the prevalence of themes studied in the literature, with the majority of studies examining the impact of FOP labeling on product reformulation (*n* = 26) and uptake (*n* = 24), and a smaller number of articles investigating nutritional comparisons between labeled and unlabeled products (*n* = 11). However, the number of studies measuring the effect of FOP labeling on food manufacturers’ practices has increased over time, as shown in Figure 3, which displays the annual number of included studies. There has been a growing interest in the analysis of FOP labels and their impact on the food environment, with a marked increase in studies from 2017 onwards.

The key findings of this review are presented in Table 3 and described in the three sections below.

### 3.1. Product Reformulation

Among the twenty-six articles evaluating the effect of FOP labels on product reformulation, two compared labeling designs, one studied summary versus positive endorsement [38] and the other nutrient-specific versus non-interpretative [55]. Furthermore, over 40% of publications assessed summary designs (*n* = 16).

Nutritionally, studies most frequently reported reductions in sodium [28,32,38,41,43,46,48,53,55,56,58,59,60,62], sugar [9,25,28,44,46,47,48,53,56,58,59], and decreases in energy content [9,25,40,46,47,48,53,56,59]. In addition, studies evaluating positive endorsement labels such as the Tick Program [53] and the Choices Logo [56,59] showed changes in fatty acids. In contrast, other studies presented minimal change [48] or did not find a statistically significant decrease [28,46].

Analyzing by labels, two studies assessed non-interpretative labels and found no effect on product reformulation [39,55]. Further, a study comparing government-targeted foods displaying the GDA versus the Traffic Light label reported that only Traffic-Light-labeled products showed a consistent sodium reduction over time [55]. All but one article [52] observed effects in reformulation associated with interpretative label implementation. Yet, the extent of reformulation varied within studies. Below are the findings for each label.

Five studies evaluated warning labels’ implementation in Chile. Three studies reported sugar reduction in sugar-sweetened beverages (over 30% mean reduction) [46,47,48], and four described sugar and calorie reduction in breakfast cereals (15% and 4% mean reduction, respectively) [9,25,46,48]. However, warning labels had a limited effect on reducing saturated fats [46,48].

The Traffic Light labels presented varied results in reformulation. In Ecuador, for instance, a study found a 13% sugar reduction in reformulated sugary drinks; however, only two of the seven drinks would have obtained a healthier score [44]. These results suggest that the reformulation could be due to a prior industry trend and not necessarily the label policy. On the other hand, two studies, one in Iran [33] and the other in the UK [20], described positive results on reformulation; however, they did not disclose the extent of the nutritional variation.

The Health Star Rating system is the summary design that has been most widely studied. Researchers have found no [31] or minor effects in reformulation, no higher than 5% sodium reduction [28,41], and from 2014 to 2016, a study found a mean of 2 kcal per 100 g energy decrease in packaged foods [40]. However, a study examining food products marketed toward children reported that after two years of Health Star Rating label implementation, all Health-Star-Rating-labeled products had been reformulated compared to 60% of non-labeled products [42].

Although limited by small sample sizes in most cases, publications about positive endorsement designs reported large reductions of nutrients of concern (i.e., 61% sodium reduction in labeled breakfast cereals) [62], sodium being the most frequently measured [32,43,53,59,60,62]. Moreover, publications evaluating the Pick the Tick program [38,43,53,60,62] and the Health Check label consistently reported sodium reduction [32].

Among the two studies that examined food manufacturers’ practices in anticipation of labeling implementation, one found that in Belgium, there was a 20% sodium and 5% sugar reduction in breakfast cereals [58]. Conversely, Chile experienced little to no changes before implementing the warning labels [36]. However, a study described that retailers removed some of their products from the market in anticipation of the UK’s Traffic Light label implementation [20]. This response is not product reformulation but could be interpreted as an early response to policy implementation.

Food manufacturers seem to respond strategically to labeling implementation regardless of the design. For example, three studies found that after one [48] and two years [9,25] of mandatory labeling implementation in Chile, manufacturers mostly reformulated products close to the nutrient thresholds for requiring a label. Firms adjusted just enough to fall below the policy cutoff. Similarly, interviews with stakeholders in Iran reported that some food products were reformulated to comply with the Traffic Light green labeling [33]. Other examples are manufacturers expressing their intentions to reformulate to qualify to carry the Tick label [38,43,53,62], and some also mentioned using it as a marketing strategy [53]. Additionally, a breakfast cereal company required reformulation so as not to affect consumer taste appeal [60]. One study described a different industry response where reformulation improved the nutritional quality of both store-branded labeled and unlabeled foods after Traffic Light label implementation in the UK [20]. This situation may be a spillover effect of the policy on other products.

### 3.2. Labeling Uptake

Among the twenty-four articles that evaluated labeling uptake, six compared labeling uptake between interpretative versus non-interpretative labels [29,35,45,50,54,55] and one contrasted the Health Star Rating with the Pick the Tick label [38]. Over half of the articles studied Health Star Rating label uptake. Most studies examined labeling in Australia (71%) and Europe (21%).

Data collection systems varied within studies. The majority of articles collected information directly from packages (*n* = 18) [20,27,28,29,30,31,34,35,37,41,42,45,50,51,54,55,57,61], three studies used self-reported information from foundations or manufacturers [49,53,56], two gathered information through a review of the literature [33,38], and one study had no information on data collection [40].

Table 3 shows that interpretative voluntary labeling systems present low uptakes, lower than 35%, regardless of their design. Nevertheless, labeling adoption increased over time in most cases [31,35,50,53,55,56,61]. The only label that did not was the Canadian Health Check logo found on less than 5% of products in 2009—10 years after implementation—and which was discontinued in 2014 [34]. Only one study examined mandatory uptake. The study found that 80% of products adhered to the Iranian Traffic Light system after two years of voluntary compliance plus two more years of enforcement [33]. Overall, food retailers were the most common users of voluntary labels, labeling products of their own brands [20,31,35,45,49,55,57]. In contrast, manufacturers’ brands had lower [31,50] and more selective uptakes [49,54], leaning towards displaying labels on products with healthier scores [27,28,29,35,37,42,50,51,55,57].

Two studies examined Health Star Rating uptake on foods directed to children. One found that by 2016, 26% of products displayed the label, and over 80% carried a healthier score [42]. Similarly, the other study observed that only 18% of products were labeled, 76% accounting for higher nutritional scores [27].

Non-interpretative labels are generally proposed by the food industry [30,54,55] and manufacturers seem to accept them more than interpretative labels. For example, after 2 years of a food industry label (GDA) and the Traffic Light system implementation in the UK, GDA’s uptake was double the Traffic Light’s (62% versus 30%) [54]. However, both labels’ presence increased over time [55]. Likewise, when the DIG label was introduced in 2006 in Australia, food manufacturers responded with rapid labeling adoption (66% increase between 2008 and 2009) [61] and high uptakes (over 60%) after 6 years of implementation [30]. Conversely, the Health Star Rating label started with low uptakes of 5 to 7% two years after its launch in 2014 [40,41]; however, its adherence increased over time to 24% in 2017 [35] and over 30% in 2019, [50] and its presence in food products seemed to be higher than non-interpretative labels [35,45,50].

Among the five studies comparing interpretative versus non-interpretative labels, four found that food products displaying the interpretative designs Health Star Rating [29,35] and Traffic Lights label [54,55] were healthier than those labeled with non-interpretative designs (energy icon and GDA label, respectively). However, one study contrasting the Health Star Rating to the DIG label described opposite results [45].

### 3.3. Nutritional Comparison between Labeled and Unlabeled Products

As shown in Table 3, all ten studies comparing nutritional composition between FOP-labeled and FOP-unlabeled products come from voluntary labeling systems. Among the two studies evaluating the Traffic Lights labeling system, one focused on nutritional changes between labeled and unlabeled foods and found improvement in both categories after the introduction of the Traffic Lights label [20]. The other study examined the likelihood of healthier products displaying a label and found a higher probability that products lower in sodium and sugar would carry the GDA and Traffic Lights labels. However, these results were only observed in products targeted by the UK government [55].

All articles studying summary designs evaluated the Health Star Rating system used in Australia and New Zealand. Nevertheless, the results were mixed. For example, two studies compared labeled and unlabeled foods directed at children [27,42]. One found that labeled foods had lower mean energy and saturated fat and higher mean protein and fiber content than unlabeled products [42], whereas the other found that labeled products were similar in energy density (ED) but had higher ED variability than unlabeled foods [27]. Similarly, a contemporaneous study found that nutrient-poor and ultra-processed foods were more likely than nutritious foods to display the label [45]. However, a study on packaged foods found that products displaying Health Star Rating labels had higher energy density but a significantly lower mean of saturated fat, total sugar and sodium, and higher fiber contents than unlabeled products [41]. In contrast, the two studies comparing nutritional quality using the Health Star Rating algorithm found that products displaying the Health Star Rating label had a higher mean score (healthier) than products not displaying the logo [35,50].

One article compared the nutritional quality of products using a positive endorsement design: the Pick the Tick label. The study found that Tick-labeled products were, on average, 14 to 76% lower in energy, saturated fat, trans fat, and sodium than non-Tick products, indicating healthier options in each food category [53].

Only one study examined a non-interpretative label design and revealed no significant nutritional difference between DIG-labeled and unlabeled breakfast cereals [39].

## 4. Discussion

Although extensive efforts have been made to understand the impacts of front-of-package labeling on consumers [15,22,63,64], this study systematically reviewed the scientific evidence in naturalistic settings on the effect of FOP label designs and enforcement styles on food manufacturers’ practices. This section summarizes the critical implications of the review for future research and policymaking.

Substantial associations between FOP labels and food manufacturers’ responses were described. The studies included in this review described different strategic industry practices according to the labeling implemented. One example of such a strategic response was manufacturers reformulating their products just below the nutrient cutoff, which avoided negative labeling [9,25,48]. Pietinen and colleagues mentioned a similar practice where, after implementing warning labeling for sodium in Finland, manufacturers reduced sodium to avoid the label [65]. Another example found in this review was that manufacturers reformulated not only to avoid a negative label but also to obtain a positive endorsement [32,38,43,53,56,59,61,62]. In addition, in voluntary systems, many firms selected to label healthier products [27,28,29,35,37,42,50,51,55,57], thus suggesting that they avoid labeling unhealthy products. That type of industry behavior might be related to the one reported by Thomson, where retailers used the Pick the Tick label as a marketing strategy [53].

This review finds that the most common reformulated nutrients were sugar and sodium [9,25,28,32,38,41,43,44,46,47,48,53,55,56,58,59,60,62]. In addition, likely as a consequence of sugar reduction, calories were significantly reduced [9,25,40,46,47,48,53,56,59]. Included studies reported these changes primarily in nutrient-specific and positive endorsement designs. It makes sense that for nutrient-specific designs, reducing nutrients of concern is more salient as food industries want to avoid negative labeling. For positive endorsement labels, the interest in reformulation comes directly from the manufacturer that wants to be perceived as a healthy brand or selling a healthy product. However, although reformulations lead to considerable nutritional improvement due to their voluntary nature, they are limited to a small sample of products. It is also worth noting that all four positive endorsement designs included in this review were discontinued by 2016.

The time of label implementation could explain the low reduction in trans fatty acids found in nutrient-specific and summary designs compared to positive endorsements. Most positive endorsement logos were implemented in the 1990s or early 2000s, many years before the other designs. During that time, additional governmental regulations on trans fat could have enhanced reformulation for those logos. In addition, fiber increase was only mentioned for summary and positive endorsement designs and not in nutrient-specific designs. This difference could be because the first two evaluate the product’s overall healthfulness and including fiber in a food product increases the product’s score. On the other hand, for nutrient-specific designs, fiber is not listed as a nutrient of concern, which could be a limitation of these designs.

Despite the mixed results found in studies evaluating the nutritional comparison between labeled and unlabeled food products, it is safe to conclude that labeled products tend to receive healthier scores than unlabeled ones when evaluated under specific nutritional criteria.

Interestingly, non-interpretative schemes, typically proposed by food companies [30,54,55], showed no effect on product reformulation [39,55]. In addition, they seem to have a greater acceptance by the industry than interpretative labels, displaying higher uptakes [30,54]. However, there was a decrease in these designs in Australia [35,45,50], possibly because the government urged food manufacturers to increase Health Star Rating adherence [50]. Food manufacturers also seemed to prefer non-interpretative versus interpretative labels when looking at a product’s nutritional quality. For example, four out of five studies found that food products displaying interpretative designs were healthier than those labeled with non-interpretative designs [29,35,54,55]. Likewise, a study described no nutritional difference between unlabeled breakfast cereals and those displaying a non-interpretative design [39]. The reductive nature of these labels could explain these results. These designs are harder to understand and do not provide decision guidance [8,66,67]; therefore, they probably do not incentivize food manufacturers to improve a product’s nutritional quality because they do not provide intuitive information to consumers. Further, these labels could motivate the manufacturers to use them in less-nutritious foods. Nevertheless, the studies evaluating these designs only examined the UK and Australia.

It is important to note that voluntary approaches not only limit consumers’ access to easy-to-understand nutritional information but also possibly limit the effects on reformulation. This review includes eight studies from three countries analyzing manufacturer’s responses to a mandatory system and it is not possible to directly compare to voluntary schemes; however, it seems likely that the response of product reformulation was more prominent when the policy was mandatory compared to voluntary. In addition, in the case of voluntary schemes, industries can choose only to label their healthier or easier-to-reformulate products, which would explain the small reformulation effect found in the literature. Indeed, many authors studying voluntary systems suggest mandatory labeling to increase their uptake [15,41,55,68].

Overall, we observed a lack of monitoring and evaluation of these types of interventions associated with industry behaviors. Even though over 30 countries have endorsed at least one FOP label, we only found 10 countries studying industry responses to FOP labeling implementation. However, it is possible that this information exists in government reports and in languages that we did not include in this review.

Our findings differ from Shangguan and colleagues’ meta-analysis in that they found significant reductions of trans fatty acids after food labeling [23], and we only found that effect from positive endorsement designs. A possible explanation for that is that their paper included back-of-package labeling, which significantly reduced TFA after mandatory disclosure policies and solid educational campaigns. Our results also differ from Shangguan’s in that we found significant sugar and calorie reductions. These differences are likely to be due to the new articles released in 2020 evaluating mandatory policies, which are likely to affect reformulation substantially. Finally, our findings coincide with Shangguan et al.’s regarding sodium reduction due to food labeling but differ from Santos et al.’s, who found no significant sodium reductions attributable to labeling interventions [69]. Similar to our difference with Shangguan et al., the years included in Santos et al.’s study could also explain this difference.

This review provides valuable information for policymakers. It found substantial real-life evidence to sustain the argument that different FOP label designs affect product reformulation and lead to good industry practices. However, food manufacturers are selectively choosing which products to label, and the effects of this on consumers are uncertain. Based on these findings, it is recommended that FOP nutrition labels are made mandatory and have an interpretative design to take advantage of the policy to its fullest, promoting product reformulation and granting consumers all the information to make informed decisions [15]. These results strengthen the available evidence regarding the positive impacts of these labels as a policy tool to address the obesity epidemic. FOP labeling has the potential to not only help guide consumers to make healthier choices but also create healthier food environments.

There are limitations to this review. One limitation is that it is not possible to conclude which FOP label design has a more substantial effect on product reformulation because two important confounders could have affected the results of the findings. The first is that all studies included in this review evaluating non-interpretative, summary or positive endorsement designs had voluntary enforcement. Only the nutrient-specific design had both types of enforcement—mandatory and voluntary. This situation makes the comparison between designs impossible. A mandatory regulation would have allowed us to compare the effect across the entire industry of pre-packaged foods and would likely have had a more considerable impact on reformulation. The second is the difficulty of disentangling the FOP labeling effect on reformulation due to the studies’ naturalistic settings. Many government policies have been implemented along with other restrictive or educational campaigns, such as marketing restrictions on labeled products or incentivizing voluntary reformulation within the food industry. Therefore, singling out the effect of FOP labeling on product reformulation is complicated. In addition, each country may have heterogeneous nutritional content starting points, so large-scale reformulation might not occur only due to labeling design, but also due to the possibility of nutritional improvement. These antecedents are critical to keep in mind when interpreting the results.

This review has several strengths. First, this review demonstrates an association between FOP labeling and the reformulation of pre-packaged foods, as well as the strategic responses of food manufacturers. This review found consistent literature to support that the food manufacturers responded to these labels by reformulating food and beverages, which reinforces the importance of implementing these labels. A second strength is that this study included a broad timeframe and key terms to include every possible available article. A third fundamental strength is the bilingual search strategy. Both reviewers were bilingual, which helped include relevant articles from Latin America, where these labels have been largely implemented. Lastly, this review covered studies evaluating interventions in real-world settings and from different parts of the world, attempting to find global patterns in true-to-life circumstances.

Future directions for this work include further research and policy advocacy. Long-term effects on reformulation should be evaluated, especially from mandatory approaches. A successful FOP labeling policy intervention would be if, after five years, the food manufacturers keep reformulating and innovating. In addition, rigorous evaluations of food manufacturers’ responses are essential to understand the effects of these labels holistically and rectify them if necessary. Furthermore, future research should measure the effect of these policies on the nutritional composition of foods in other countries (spillover effects). It was not within the scope of this review to include the unintended consequences of these policies. However, it is critical to monitor any compensatory measures of reformulation, such as overuse and intake of non-nutritive sweeteners (NNS) given the uncertainty about their relationship with health outcomes [70]. In addition, the information gathered should help advocacy groups and public health professionals to disseminate and advocate for the implementation of FOP labels in countries where this policy does not yet exist. It should also benefit countries where FOP labeling is voluntary to consider the benefits of mandatory regulations. Product reformulation holds promise in reducing the population’s intake of nutrients of concern [4,65].

## 5. Conclusions

In conclusion, there is evidence that front-of-package nutrition label implementation is associated with food manufacturers’ responses. It appeared that these responses were heterogeneous and depended on label designs and types of enforcement. Moreover, interpretative labels were indicated to be better at encouraging product reformulation, whereas non-interpretative labels did not. However, voluntary labels showed low uptakes and food manufacturers showed a preference to label healthier products.

It was not possible to make conclusions about which FOP label design type had a more substantial effect on reformulation because only nutrient-specific designs had both mandatory and voluntary enforcement. However, it is suggested that policymakers take a mandatory approach regardless of labeling design, as it can significantly impact the food environment.

## Figures and Tables

**Table 1:** Summary of studies included by geographic location, FOP label design and enforcement style.

	Number of Studies	Countries		FOP Label Design			Enforcement Style	
			Non-Interpretative	Nutrient-Specific	Summary	Positive Endorsement	Mandatory	Voluntary
South America	7 (18%)	Chile (6), Ecuador (1)	-	7	-	-	7	-
North America	3 (8%)	Canada (2) USA (1)	-	-	-	3	-	3
Europe	7 (18%)	UK (3), Belgium (2), The Netherlands (2)	2	3	2	2	-	9
Asia	1 (2%)	Iran (1)	-	1	-	-	1	-
Oceania	21 (54%)	Australia (16) New Zealand (6)	6	-	14	5	-	25
Total *	39	10	8	11	16	10	8	37

* Some studies compared more than one country or labeling design.

**Table 2:** Dimensions of FOP labeling included in the review.

Design Classification	Type	Label Image	Country	Enforcement	Institution
Non-interpretative	Guideline Daily Amount (GDA)	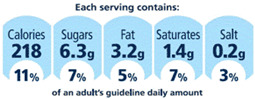	UK	Voluntary	Food Industry
	Daily Intake Guide (DIG)	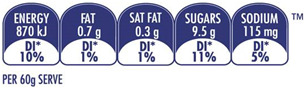	Australia	Voluntary	Food Industry
	Energy Icon	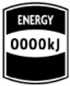	Australia	Voluntary	Government
Nutrient specific	Warning Labels	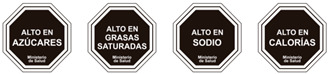	Chile	Mandatory	Government
	Traffic Lights	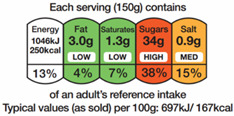	UK	Voluntary	Government
	Traffic Lights	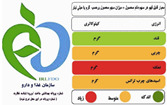	Iran	Mandatory	Government
	Traffic Lights	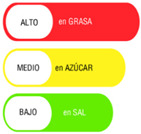	Ecuador	Mandatory	Government
Summary	Nutri-Score	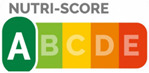	Belgium	Voluntary	Government
	Health Star Rating	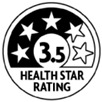	Australia and New Zealand	Voluntary	Government
Positive endorsement	Pick the Tick	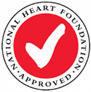	Australia and New Zealand	Voluntary	NGO
	Choices Logo	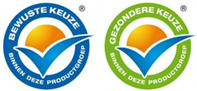	The Netherlands	Voluntary	Food Industry
	Health Check	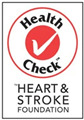	Canada	Voluntary	NGO
	Walmart initiative	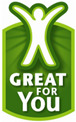	USA	Voluntary	Food Industry

**Table 3:** Effects on food manufacturers’ practices by FOP label design and type of enforcement.

Author, Year	Country, Label Names, Year of Implementation	Sample	Thematic Findings	Key Findings
Alé-Chilet, 2021 [25]	Chile Warning Labels, 2016	Breakfast cereal market (*n* = 131). 2016 to 2018.	Reformulation	Labeling implementation along with associated marketing restrictions induced a mean of 4% calorie and 8% sugar reduction of product nutritional content.
Azzopardi, 2020 [27]	Australia Health Star Rating, 2014	Foods directed to children 5–12. (*n* = 548) Beverages excluded. 2014 to 2018	Uptake Nutritional comparison	18.2% of products were labeled. Bakery, Breakfast Cereals, and Snacks accounted for 80% of all labeled items. 76% of labeled products had a high HSR score (healthier). Similar energy content between labeled and un-labeled products (356 kcal/100 g vs. 381 kcal/100 g)
Bablani, 2020 [28]	Australia & New Zealand Health Star Rating, 2014	Non-seasonal packaged unique products. Products with the HSR energy-only icon logos excluded. (*n* = 58,905) 2013 to 2019.	Uptake Reformulation	Low-scored (unhealthier) products were less likely to adopt HSR compared to high-scored (healthier) ones (<15%, over 35%, respectively) 4% sodium reduction in NZ and 2% in Australia; and 2% sugar reduction in NZ. No change in protein or saturated fat content. Greater reformulation for initially unhealthy products.
Barahona, 2020 [9]	Chile Warning Labels, 2016	Breakfast cereal (*n* = 94). 2016 to 2018.	Reformulation	Sugar and calories reduction (12% and 3%, respectively after two years of policy implementation). Over 60% of these products, reformulated to fall right below the mandated threshold, thus avoiding the label.
Brownbill, 2019 [29]	Australia Health Star Rating, 2014 Energy icon, 2014	Ready-to-drink (≤600 mL) non-dairy/non-alcoholic beverages (*n* = 762). 2016	Uptake	35% of beverages carried a FOP label; 28% displayed the energy-only icon and 7% the HSR label. Products carrying the star rating icon rated higher (healthier) being predominantly on 100% fruit juices (85.7%). Most star rating labeled beverages contained high amounts of sugar.
Carter, 2013 [30]	Australia Daily Intake Guide, 2006	Packaged foods energy-dense but nutrient-poor (*n* = 728). 2012.	Uptake	The 66% of energy-dense nutrient-poor products carried the label.
Castro, 2021 [31]	New Zealand Health Star Rating, 2014	Store-brands (*n* = 4266) branded (*n* = 19,318) food products across 21 food categories. 2015–2019.	Uptake Reformulation	By 2019, 92% of store-brand products displayed the HSR on the package compared with 17% on branded food products. During the study period, there was an increase in product labeling overall. No consistent changes in sodium or sugar contents.
Dummer, 2012 [32]	Canada Health Check, 1999	Health Check program licensees’ products (*n* = 371). 2009	Reformulation	One-hundred fifty labeled products self-reported to have reduced sodium before obtaining the label.
Edalati, 2020 [33]	Iran Traffic Light label, 2016	Food products Date and sample number not reported.	Uptake Reformulation	An 80% of food products carried the traffic light label in 2018, when it became mandatory. Self-reported reformulation to comply with green labelling requirements in some products. Interviewees mentioned perceived increased fraud to obtain green labelling and addition of non-caloric sweeteners to replace sugars.
Elliot, 2019 [34]	Canada Health Check, 1999	Child- directed food products. 2009 (*n* = 354) 2017 (*n* = 374).	Uptake	Only 5% of products were labeled with the Health Check in 2009 and by 2017 the label had been discontinued.
Fichera, 2020 [20]	UK Traffic Light label, 2006	Store-brands and branded foods (*n* = 360,921) purchased by 20,707 households. 2005–2008.	Uptake Nutritional comparison Reformulation	Label was found on store-branded food of four food retailers in the UK. After labeling implementation, nutritional improvement in labeled and unlabeled store-branded foods. Retailers brought forward the time to discontinue some products to take place before labelling introduction.
Jones, 2018 [35]	Australia Health Star Rating, 2014 Energy icon, 2014	Packaged foods (*n* = 15,767) 2014–2017	Uptake Nutritional comparison	By 2017, 28% of products were labeled: 24% HSR and 4% energy icon only. A linear increase in uptake from 2014 to 2017. 76% of labeled products had high scores (healthier). 77% of products labeled with the energy icon had a low score (unhealthy). Most products were confectionery foods and non-alcoholic beverages. Labeled products had a higher mean HSR score (healthier) than unlabeled products. (unhealthier).
Kanter, 2019 [36]	Chile Warning Labels, 2016	Packaged food and beverage products in 2015 (*n* = 5421) 2016 (*n* = 5479).	Reformulation	In preparation for label implementation, less than 5% reductions on targeted nutrients and caloric content and increments in nutrient of concern in some products.
Lawrence, 2018 [37]	Australia Health Star Rating, 2014	New food products (*n* = 12,108) 2014–2017	Uptake	10% of the sample displayed the HSR. Majority of labeled products displayed a “healthier” score. More than half foods categorized as ‘non-nutritious” presented an HSR-high score (healthier).
Lindberg, 2017 [38]	Australia Pick the Tick, 1997 Health Star Rating, 2014	Food products (*n* = 33 manufacturers self-report). 2010–2017	Uptake Reformulation	Two manufacturers disclosed using the HSR label, and four used the Tick label. Self-reports indicated that Tick participation led four manufacturers to reduce salt content and one due to the HSR.
Louie, 2012 [39]	Australia Daily Intake Guide, 2006	Breakfast cereals. 2004 (*n* = 128) 2010 (*n* = 197)	Nutritional comparison Reformulation	By 2010, no significant difference in nutritional composition between DIG-labeled and non-labeled. No significant difference in the nutritional composition of breakfast cereals during the study period. No product reformulation after label implementation.
Mantilla-Herrera et al., 2018 [40]	Australia Health Star Rating, 2014	Pre-packaged food and beverage products available in both 2013 and 2016 (*n* = 14,986).	Uptake Reformulation	7% of the sample carried the HSR in 2016. Labeled products reduced a mean of 2 kcal per 100 g.
Mhurchu, 2017 [41]	NZ Health Star Rating, 2014	Matched food and beverages (*n* = 15,357). 2014–2016.	Uptake Nutritional comparison Reformulation	By 2016, 5% of packaged food and beverage displayed the HSR label. Higher uptakes on cereals, convenience foods, packaged fruit and vegetables, and sauces and spreads. Labeled products had higher energy density but lower saturated fat, total sugar, and sodium than unlabeled products. Greater reformulation of HSR-labeled products compared to non-labeled products. Sodium content of labeled products decreased by 5%, and sodium unlabeled increased a 3% in unlabeled products.
Morrison, 2019 [42]	Australia Health Star Rating, 2014	Packaged food products marketed towards children 2013 (*n* = 156) 2016 (*n* = 252)	Uptake Nutritional comparison Reformulation	26% of products displayed HSR label. Over 80% displayed a high score (healthier). Labeled products had lower mean energy and saturated fat content and higher mean protein and fiber content than non-HSR labeled products. All labeled products in 2013 were reformulated by 2016, compared to 61% of non-HSR labeled products.
Ning, 2017 [43]	NZ Pick the Tick, 1991	Breakfast cereals, edible spreads, processed poultry, cooking sauces. (*n* = 52). 2011–2013.	Reformulation	36% of products were formulated and reformulated. 46% mean sodium reduction. Breakfast cereals had the highest reformulation (59%). Manufacturers said that sodium reduction was, in addition to other drivers, influenced by the Pick the Tick program.
Peñaherrera, 2019 [44]	Ecuador Traffic Light Labels, 2014	Soft drinks (*n* = 14 brands) 2013 to 2015	Reformulation	50% of soft drink brands reduced sugar (13% mean sugar reduction). However, of those, only 29% of products (2 brands) led to a yellow or green light change (healthier score).
Pulker, 2018 [45]	Australia Daily Intake Guide, 2006 Health Star Rating, 2014	Supermarket own brand foods (*n* = 3940) 2017	Uptake Nutritional comparison	81.5% of products were labeled. No products included both labels. Over half displayed the HSR label and a quarter the DIG label. Nutrient-poor and ultra-processed foods were more likely than nutritious foods (vegetables, legumes, etc) to display the HSR label.
Quintiliano Scarpelli, 2020 [46]	Chile Warning Labels, 2016	Packaged foods and beverages 2013 (*n* = 551) 2019 (*n* = 476)	Reformulation	Overall, sugar decreased by 15%. Over 50% sugar reduction in dairy, confitures, and sugary beverages. Energy reduction in flour-based foods, confitures, fats and oils, dairy and sugary drinks. Sodium reduction in fats and oils and spices (41%), condiments, and sauces (38%). Little reformulation in pastry, desserts and ice creams. Not significant changes on saturated fats.
Quitral, 2019 [47]	Chile Warning Labels, 2016	Fruit juices and soft drinks. T0 periods prior to labeling implementation (month/year not reported) T1 2017 (*n* = 7)	Reformulation	78% mean energy and 96% mean sugar content reduction. 128% increase in non-caloric sweeteners content. However, the study does not report statistical significance.
Reyes, 2020 [48]	Chile Warning Labels, 2016	Packaged foods and beverages. 2015 or 2016 (*n* = 4055) 2017 (*n* = 3025).	Reformulation	Overall decrease in products displaying warning labels (from 51% to 44%). Most reformulated products fall right below the mandated threshold, thus avoiding the label. Beverages, milk-based drinks, breakfast cereals, sweet baked products, and spreads products carrying a sugar warning label decreased from 80% to 60%. Savory spreads, cheeses, ready-to-eat meals, soups, and sausages products labeled as high in sodium reduced from 74% to 27%. Savory spreads and breakfast cereals labeled as calories warning label decreased 38% and 25%, respectively. Limited change in saturated fat warning label appearance: savory spreads only (38% reduction of products)
Sacks, 2020 [49]	Australia Health Star Rating, 2014	Companies operating in Australia. (*n* = 34) 2018	Uptake	Over 50% of manufacturers publicly committed to label all or some of their products. Two large supermarkets committed to their full product range. No label on added sugars or trans fat.
Shahid, 2020 [50]	Australia Health Star Rating, 2014 Energy icon, 2014	15 food categories of eligible products (*n* = 17,477). 2014 to 2019	Uptake Nutritional comparison	After 5 years of label implementation, a third of products displayed the HSR logo, and less than 10% the energy icon only. HSR label uptake had a linear annual increase of 7% since 2014. Higher labeling uptake by retailers than by manufacturers. More than three quarters of labeled products had high scores (healthier). Over 50% of fish and fish products, fruit and vegetables, and convenience foods, carried the HSR label. Under 30% of sugars, oils and sauces were labeled. Healthier products displaying the HSR logo compared to those not carrying the logo or displaying the energy icon only.
Shi, 2018 [51]	Australia Health Star Rating, 2014	Packaged foods in vending machines. 2014 (*n* = 61 vending machines; 1836 slots. 2017 (*n* = 71 vending machines; 2458 slots)	Uptake	Under 10% of packaged food and beverages were labeled in 2017, and all of them received a high score (healthier).
Taillie, LS, Ng, SW, & Popkin, BM, 2015 [52]	USA Walmart initiative, 2011	Households’ packaged food purchases Walmart (*n* = 1,212,803) Other chain retailers (*n* = 2,521,128). 2000 to 2013	Reformulation	Compared to other chains, Walmart packaged food purchases had a greater reduction on energy density, total sugar, and sodium during the study period compared other chains. However, labeling did not seem to influence reformulation based on previous trends.
Thomson, 2016 [53]	New Zealand Pick the Tick, 1991	Newly licensed Tick products from five food categories Edible Oil Spreads, Yoghurt & Dairy Desserts, Frozen Desserts, Ready Meals and Processed Poultry. (*n* = 45). 2011 to 2013.	Uptake Nutritional comparison Reformulation	Manufacturers self-reported consumer demands influenced Tick product development and sales. The label was used as a marketing strategy. Encouraged energy, saturated fat, trans fat, and sodium reductions. Tick products were 14% to 76% lower in energy, saturated fat, trans fat and sodium than non-Tick products. In 2017, the proportion of healthy snacks and beverages increased from 7 to 14% and 38 to 44% since 2014, respectively.
Van Camp, 2010 [54]	UK Guideline Daily Amount, 2005 Traffic Light labels, 2006	Food and drinks released in the UK (*n* = 27,004) 2002 to 2008.	Uptake	GDA labeling was higher on “target” products designated by the UK government and “non-target” product as compared to the Traffic Light System (TLS) (42% vs. 26% and 20% vs. 4%, respectively) in 2008. TLS and GDA label use varies depending on company and food category.
Van Camp, 2012 [55]	UK Guideline Daily Amount, 2005 Traffic Light labels, 2006	Bread, cakes, cereal, meat products, pastries, pizzas, prepared meals, sandwiches, crackers, salty snacks and cookies (*n* = 2201) 2007 to 2009.	Uptake Nutritional comparison Reformulation	TLS labels mostly present on “target” products designated by the UK government and branded products. Labeling increased over time. Products lower in sodium and sugar were more likely to carry both GDA and TLS. For meat and prepared meals, lower sodium and saturated fat showed higher odds to use TLS as compared to GDA. Sodium reduction trend over time on “target” TLS-labeled products.
van der Bend, 2020 [56]	The Netherlands Choices Logo, 2006	Products, including 27 basic and non-basic product (sub) categories (*n* = 4343). 2006 to 2016.	Uptake Reformulation	Labeled products increased 161% over time. Reformulation varied by food category and nutrient. Trans fat and sodium were most likely to reduce as well as to have higher reductions. Energy density, saturated fat, and sugar reduction and fiber increase in half of food categories. Changes in added sugar content were not consistent over time. Saturated fat decreased by 18% and trans fat content by 48% in all products.
Vandevijvere, 2020 [57]	Belgium Nutri-Score, 2018	Food products (*n* = 1781). 2019	Uptake	10% of products on the market in Belgium displayed the NS. About 90% of them were own-brand products from two major food retailers. About 56% of products displayed a healthy score while 26% of products displayed an unhealthy score.
Vermote, 2020 [58]	Belgium Nutri-Score, 2018	Breakfast cereals (*n* = 275) 2017 and 2018.	Reformulation	Reformulation in anticipation of policy implementation: 3% fiber and 2% protein increase. 5% sugar and 20% sodium reduction. A or B scored products (healthier): 34% (2017) versus 37% (2018) D or E scored products (unhealthier): 22% (2017) versus 20% (2018).
Vyth, 2010 [59]	The Netherlands Choices Logo, 2006	Fruit juices, processed meats, dairies, sandwiches, soups, sauces, sandwich fillings (*n* = 821). 2007 to 2009.	Reformulation	20% of products were reformulated. 29% of products were newly developed. Reformulation and new product development mostly on soups and snacks. Caloric content reduction in dairy products, sandwich fillings (10% and 30%, respectively). Sugar reduction in dairy products and sauces (75% and 13%, respectively). Saturated fats reductions in meat and dairy products (43% and 30%, respectively). Sodium reduction was the most common change found in processed meats, sandwiches, soups and sandwich fillings (18%, 42%, 13%, 39%, respectively). 51% fiber increase in sandwiches. Newly developed sandwiches, had over 500% higher sugar than reference sandwiches, as well as 33% higher fiber.
Williams, 2003 [60]	Australia Pick the Tick, 1997	Kellogg’s Breakfast cereals. 1997 (*n* = 12)	Reformulation	Two-thirds of the total breakfast cereals sales volume reduced sodium. Sodium reduction varied by product. Manufacturer required that reformulation did not affect consumer taste appeal. 40% mean sodium reduction (from 12% to 88%) 42% of reformulated products were eligible to be labeled.
Williams, 2010 [61]	Australia Daily Intake Guide, 2006	Products at supermarkets. (*n* = not reported) 2007, 2008 and 2009.	Uptake	Labeling increased over time (66% in the six months between the last two surveys in 2009). 60% of products carried the energy only label 40% displayed energy plus additional nutrients. DIG mostly present in biscuits and crackers, cooking sauces, breakfast cereals, ice cream, soft drinks, processed meats, frozen foods, snack foods and juices and confectionery. Based on researchers’ estimation about 10% of products carried the DIG label in late 2009.
Young, 2002 [62]	New Zealand Pick the Tick, 1991	Reformulated or formulated bread, breakfast cereals and margarine from companies participating in the Pick the Tick program. (*n* = 23). 1998 to 1999	Reformulation	Sodium reduction in breakfast cereals by 61%, bread by 26%, and margarine by 11%. Manufacturers expressed that sodium changes were made to qualify to carry the label.

## Data Availability

No new data were created or analyzed in this study. Data sharing is not applicable to this article.

## Appendix A. Search Protocol

Questions	What is the effect of front-of-package labels on food manufacturers’ practices? What effect do different front-of-package label designs have on product reformulation? What effect do different enforcement styles have on food manufacturers’ practices?	
Search Strategy	Database Sources	PubMed → 1980 results Public Affairs Information Service International (PAIS Index) →367 results Cochrane → 2 Reviews, 370 Trials SCOPUS → 7586 results ABI/Inform (Business and Management) → 1912 results. After excluding magazines and newspapers → 1280 results. Only retrieved the first 1000 more relevant due to site restrictions. Google Scholar Multiple database search using EBSCO → 6574 results Spanish database Scielo → 154 results added extra Spanish words Total:
	Search Date & Terms	Search date: 1 September 2020 Second date: 9 May 2021 Third date: 28 May 2021 Key Terms: “Nutrition label*” “Nutrition logo” “Front of Pack*” “Food label*” “Warning Label*” Extra Key terms Spanish databases: “Logo nutricional” “Etiquetado nutricional”
	Ancestry Search	References of articles Grey literature Working papers
Study Selection & Rating	Inclusion Criteria	Natural and quasi-experimental studies that evaluate modifications to the nutritional characteristics of the food and beverage supply after a government or non-government organization implemented a standardized interpretative FOP label or labelling uptake. Both voluntary and mandatory approaches included. The years included in the search included 1 January 1990, to 31 August 2020 Outcomes of interest: Product reformulation before and after labelling implementation. Nutrient-specific changes. Labelling uptake Nutritional comparison between labeled and unlabeled products English & Spanish language only.
	Exclusion Criteria	FOP labeling referred to non-nutrient-based claims such as Organic, GMO, country of origin; product-specific industry claims; health or nutrition claims. Non-interpretative FOP label (monochrome numerical information). FOP for alcohol. No front-of-pack nutrition labels. Abstract or PDF not available
	Primary Screening	One person screening of titles and abstracts for relevance
	Secondary Screening	Two people screening of full articles for relevance

## Appendix B. Methodology—Search Strategy and Key MESH Terms Included

PubMed: ((((“Nutrition label*”[Title/Abstract]) OR (“Nutrition logo”[Title/Abstract])) OR (“Front of Pack*”[Title/Abstract])) OR (“Food label*”[Title/Abstract])) OR (“Warning Label*”[Title/Abstract])

PAIS index: ab((“nutrition labeling” OR “nutrition labels”)) OR ab(“Nutrition logo”) OR ab(“Front of Pack*”) OR ab((“food label” OR “food labeling” OR “food labelling” OR “food labels”)) OR ab((“warning label” OR “warning labels”))

Scielo: (ab:(“Nutrition label”)) OR (ab:(“Nutrition logo”)) OR (ab:(“Front of Pack”)) OR (ab:(“Food label”)) OR (ab:(“Warning Label”)) OR (ab:(“logo nutricion”)) OR (ab:(“etiquetado nutricional”))

Scopus: (TITLE-ABS-KEY (“Nutrition label*”) OR TITLE-ABS-KEY (“Nutrition logo”) OR TITLE-ABS-KEY (“Front of Pack*”) OR TITLE-ABS-KEY (“Food label*”) OR TITLE-ABS-KEY (“Warning Label*”)) AND PUBYEAR > 1989 AND (LIMIT-TO (LANGUAGE, “English”) OR LIMIT-TO (LANGUAGE, “Spanish”))

Cochrane: “Nutrition label*” in Abstract OR “Nutrition logo” in Abstract OR “Front of Pack*” in Abstract OR “Food label*” in Abstract OR “Warning Label*” in Abstract—with Cochrane Library publication date Between Jan 1990 and Jan 2020 (Word variations have been searched)

EBSCO Host: AB “Nutrition label*” OR AB “Nutrition logo*” OR AB “Front of Pack*”

Limiters—Publication Date: 19900101-20201231; Edition: London

Expanders—Apply equivalent subjects

Narrow by Language:—spanish

Narrow by Language:—english

Search modes—Find all my search terms
